# Changes in total choline concentration in the breast of healthy fertile young women in relation to menstrual cycle or use of oral contraceptives: a 3-T ^1^H-MRS study

**DOI:** 10.1186/s41747-018-0075-0

**Published:** 2018-12-17

**Authors:** Giovanni Di Leo, Ileana Ioan, Maria Laura Luciani, Cecilia Midulla, Franca Podo, Francesco Sardanelli, Federica Pediconi

**Affiliations:** 1 Radiology Unit, IRCCS Policlinico San Donato, San Donato Milanese, Italy; 2 Postgraduate School in Radiodiagnostics, Università degli Studi di Milano, Milan, Italy; 3 Department of Radiological, Oncological and Pathological Sciences, Sapienza University of Rome, Rome, Italy; 4 Department of Cell Biology and Neurosciences, Istituto Superiore di Sanità, Rome, Italy; 5 Department of Biomedical Sciences for Health, Università degli Studi di Milano, San Donato Milanese, Italy

**Keywords:** Breast, Choline, Contraceptives (oral), Magnetic resonance spectroscopy, Menstrual cycle

## Abstract

**Background:**

To evaluate changes in total choline (tCho) absolute concentration ([tCho]) in the breast of healthy fertile women in relation to menstrual cycle (MC) or use of oral contraceptives (OC).

**Methods:**

After institutional review board approval, we prospectively evaluated 40 healthy fertile volunteers: 20 with physiological MC, aged 28 ± 3 years (mean ± standard deviation; nOC group); 20 using OC, aged 26 ± 3 years (OC group). Hormonal assays and water-suppressed single-voxel 3-T proton magnetic resonance spectroscopy (^1^H-MRS) were performed on MC days 7, 14, and 21 in the nOC group and only on MC day 14 in the OC group. [tCho] was measured versus an external phantom. Mann-Whitney *U* test and Spearman coefficient were used; data are given as median and interquartile interval.

**Results:**

All spectra had good quality. In the nOC group, [tCho] (mM) did not change significantly during MC: 0.8 (0.3–2.4) on day 7, 0.9 (0.4–1.2) on day 14, and 0.4 (0.2–0.8) on day 21 (*p* = 0.963). In the OC group, [tCho] was 0.7 (0.2–1.7) mM. The between-groups difference was not significant on all days (*p* ≥ 0.411). All hormones except prolactin changed during MC (*p* ≤ 0.024). In the OC group, [tCho] showed a borderline correlation with estradiol (*r* = 0.458, *p* = 0.056), but no correlation with other hormones (*p* ≥ 0.128). In the nOC group, [tCho] negatively correlated with prolactin (*r* = -0.587, *p* = 0.006) on day 7; positive correlation was found with estradiol on day 14 (*r* = 0.679, *p* = 0.001).

**Conclusions:**

A tCho peak can be detected in the normal mammary gland using 3-T ^1^H-MRS. The [tCho] in healthy volunteers was 0.4–0.9 mM, constant over the MC and independent of OC use.

## Key points

- Using 3-T magnetic resonance spectroscopy, a total choline peak may be detected in normal breast tissue.
- Absolute total choline concentration does not change with menstrual cycle when not using contraceptives.
- Use of oral contraceptives does not impact on absolute total choline concentration.
- No clear correlation exists between absolute total choline concentration and sex hormones.

## Background

Proton magnetic resonance spectroscopy (^1^H-MRS) allows non-invasive metabolic imaging analyses of biologic tissues and organs, including the human breast [1–5], and may increase the positive predictive value of magnetic resonance imaging (MRI) when used for breast cancer detection [3, 6, 7]. Systematic reviews of ^1^H-MRS of breast lesions have shown a pooled sensitivity between 71% and 74% and a pooled specificity between 78% and 88% [8–12].

The role of ^1^H-MRS in breast examination is typically based on the detection of the so-called total-choline (tCho) peak, which is mainly the result of resonances between 3.14 and 3.34 ppm arising from the trimethylammonium headgroups of water-soluble choline-containing metabolites, i.e., glycerophosphocholine, phosphocholine, and free choline [12]. Increased tCho concentrations ([tCho]) up to 2.2 mM (range of 0.0–8.5 mM) have been reported at 4.0 T in cancer lesions [2] and are mostly attributed to increases in phosphocholine as a consequence of increased metabolic turnover of phospholipid precursors and derivatives. Using optimised pre-processing, Stanwell et al. [13] have also shown the capability at 1.5 T to distinguish phosphocholine resonance from the tCho large peak, although this result was not confirmed by other authors.

The value of tCho-based MRS as an early predictor of tumour response to neoadjuvant chemotherapy has been proven. A 2009 1.5-T ^1^H-MRS study by Baek et al*.* [14] assessed 35 breast cancer patients from before neoadjuvant chemotherapy to the first follow-up assessment after one to two cycles of doxorubicin and cyclophosphamide and a second assessment after another two cycles. The authors reported that, at second follow-up, patients with a pathologically complete response had a significantly greater reduction in [tCho] relative to the change in tumour size than those who had an incomplete response.

Little is still known about the tCho levels in normal mammary gland. According to studies on excised human tissues and cell cultures, the biochemical profile of normal gland is considered similar to that of benign lesions, in which substantially lower tCho levels are usually detected compared to cancer lesions [15].

During the menstrual cycle, breast tissues undergo changes, including proliferation, differentiation, and regression [16]. These changes were demonstrated to produce variations of background parenchymal enhancement on contrast-enhanced MRI [17, 18]. In particular, background enhancement is lower in the follicular than in the luteal phase [19]. Thus, it is highly recommended to schedule contrast-enhanced breast MRI during the follicular phase (from day 5 to day 12 after the start of the menstrual cycle) to avoid an assumed risk of false-positive diagnoses [17]. Importantly, no changes in MRI parameters have been reported in young women using oral contraceptives (OC) [20].

As tCho may be used as a biomarker of breast cancer, it is important to assess whether its ^1^H-MRS resonance normally changes during the menstrual cycle, as these changes could act as a confounding factor in diagnosis. Moreover, it is also important to evaluate the effect of OC use on the tCho level in the normal breast.

Thus, our aims were: 1) to measure [tCho] in the normal mammary gland of young healthy women and to determine whether it varies during the menstrual cycle; and 2) to compare women not using OC with those using OC.

## Methods

### Study design and population

This prospective longitudinal study was approved by our institutional review board and written informed consent was obtained from all participants.

A total of 40 healthy fertile volunteers without family history of breast or ovarian cancer entered this study. All women underwent initial clinical examination and breast ultrasound to check for compliance with inclusion criteria. Exclusion criteria were: previous breast surgery, recent breast feeding, presence of breast nodules or cysts larger than 1 cm, and contraindications to undergo MRI.

Volunteers were divided into two groups according to whether they had a physiological menstrual cycle or they used OC, as follows: a group called “nOC” included 20 women with physiological menstrual cycle, aged 28 ± 3 years (mean ± standard deviation; median 27); a group called “OC” included 20 women using OC, aged 26 ± 3 years (median 26). The mean body mass index ± standard deviation was 21 ± 3 kg/m^2^ for the nOC group and 20 ± 2 kg/m^2^ for the OC group; the mean age at menarche ± standard deviation was 12 ± 1 year and 13 ± 1 year, respectively.

### Hormonal assay

Each participant underwent a hormonal assay using a radioimmunoassay technique based on labeled antigen–antibody binding [21]. Estradiol, progesterone, follicle stimulating hormone, luteinising hormone, and prolactin were measured. Women in the nOC group underwent hormonal assay three times, on days 7, 14, and 21 of the menstrual cycle; women in the OC group underwent hormonal assay only once, on day 14.

### ^1^H-MRS protocol

As for the hormonal assay, women in the nOC group underwent ^1^H MRS three times, on days 7, 14, and 21 of the menstrual cycle; women in the OC group underwent ^1^H MRS only once, on day 14.

**Fig. 1:**
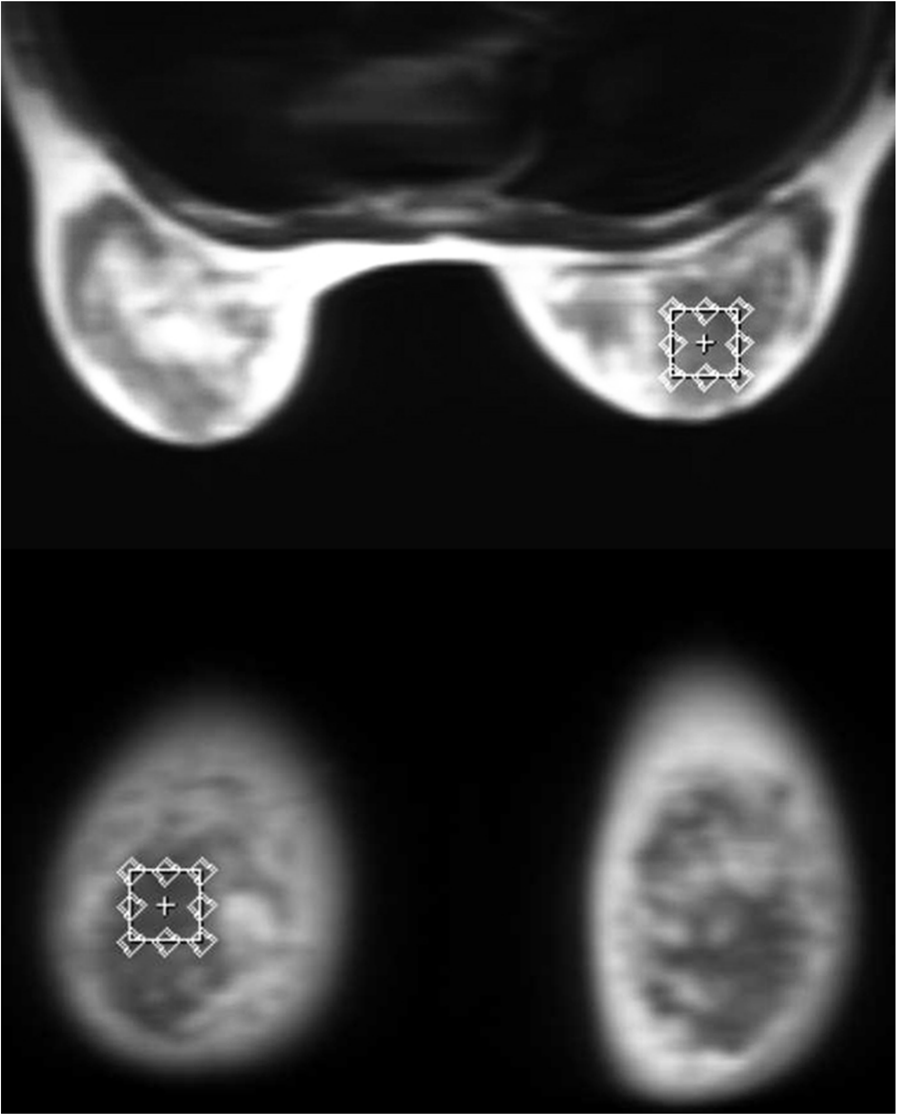
Placement of the volume of interest for spectrum acquisition into the most homogeneous part of the gland

All MRS studies were performed using a 3-T unit (General Electric Healthcare, Discovery MR 750, Little Chalfont, UK). Automated shimming was performed before each examination. Water suppressed single-voxel MRS was acquired using a point-resolved MRS sequence (repetition time 1,500 ms, echo time 135 ms, number of excitations 82) and a bilateral dedicated eight-channel breast coil with the patient in the prone position. A repetition time of 1,500 ms was chosen to shorten the acquisition time and avoid participants’ movement. An isotropic voxel of 2 × 2 × 2 cm^3^ was placed on the most homogeneous part of the mammary gland, thus avoiding partial-volume effects (Fig. 1).

All spectra were processed by a physicist with 5 years of experience using the software provided by the manufacturer (SAGE, v07, General Electric Healthcare, Little Chalfont, UK). After filtering and baseline and phase correction, the peak amplitude of tCho (centred at about 3.2 ppm) was measured in arbitrary units (au).

The formula by Bolan [22] was used to correct the peak amplitude of tCho (*tCho*^'^):

$$ {tCho}^{\hbox{'}}=\frac{tCho}{f_{gain}{f}_{coil}{f}_{T_1}{f}_{T_2}} $$

with correction factors:

$$ {f}_{gain}= gain/{gain}_0 $$

$$ {f}_{coil}={B}_1/{B}_{1,0} $$

$$ {f}_{T_1}\approx 1-{e}^{-\frac{TR}{T_1}} $$

$$ {f}_{T_2}={e}^{-\frac{TE}{T_2}} $$

where *gain* is the receiver gain and B_1_ is the local amplitude of the excitation radiofrequency field, both retrieved from DICOM metadata. T1 and T2 relaxation times of choline were taken from the literature as being 870 ms and 400 ms, respectively. After these corrections are made, the signal amplitude is proportional to the number of nuclei in the volume [22].

To quantify the absolute [tCho], expressed as mM, the same MRS protocol and the same fitting procedure were applied once to a commercial phantom containing choline at a known concentration of 2 mM. In general, the corrected signal amplitude *A*^'^ of a resonance is proportional to the number of nuclei n in the sample: *n* = *k*_*sys*_*A*^'^. The system constant *k*_*sys*_ accounts for the system-specific hardware and software. The externally referenced [tCho] was then expressed as:

$$ \left[\mathrm{tCho}\right]=\frac{tCho}{f_{gain}{f}_{coil}{f}_{T_1}{f}_{T_2}}\frac{k_{sys}}{\rho_{ph}} $$

where ρ_ph_ was the phantom density, assumed to be 1 kg/L.

#### Statistical analysis

The statistical analysis was performed using the SPSS software package (SPSS IBM inc. v19, Chicago, IL, USA). A *p* value < 0.05 was regarded as significant.

Considering the small sample size, continuous tCho data were reported as median and interquartile interval (25th–75th percentile) and non-parametric statistics were adopted.

Age, body mass index, and age at menarche were presented as mean ± standard deviation; differences between the two groups were ascertained using the Student *t* test.

For the nOC group, changes over the menstrual cycle of [tCho] and hormones were assessed using the Friedman test. The [tCho] measured in the OC group was compared to [tCho] measured in the nOC group at days 7, 14, and 21 of the menstrual cycle using the Mann-Whitney *U* test. In both groups, [tCho] was correlated to concentration of hormones using the Spearman correlation coefficient.

## Results

### Distributions and associations

The two groups were homogeneous in terms of mean age and body mass index (*p* ≥ 0.250) but women in the nOC group had a slightly lower age at menarche than women in the OC group (12 ± 1 years versus 13 ± 1 years, *p* = 0.028).

**Fig. 2:**
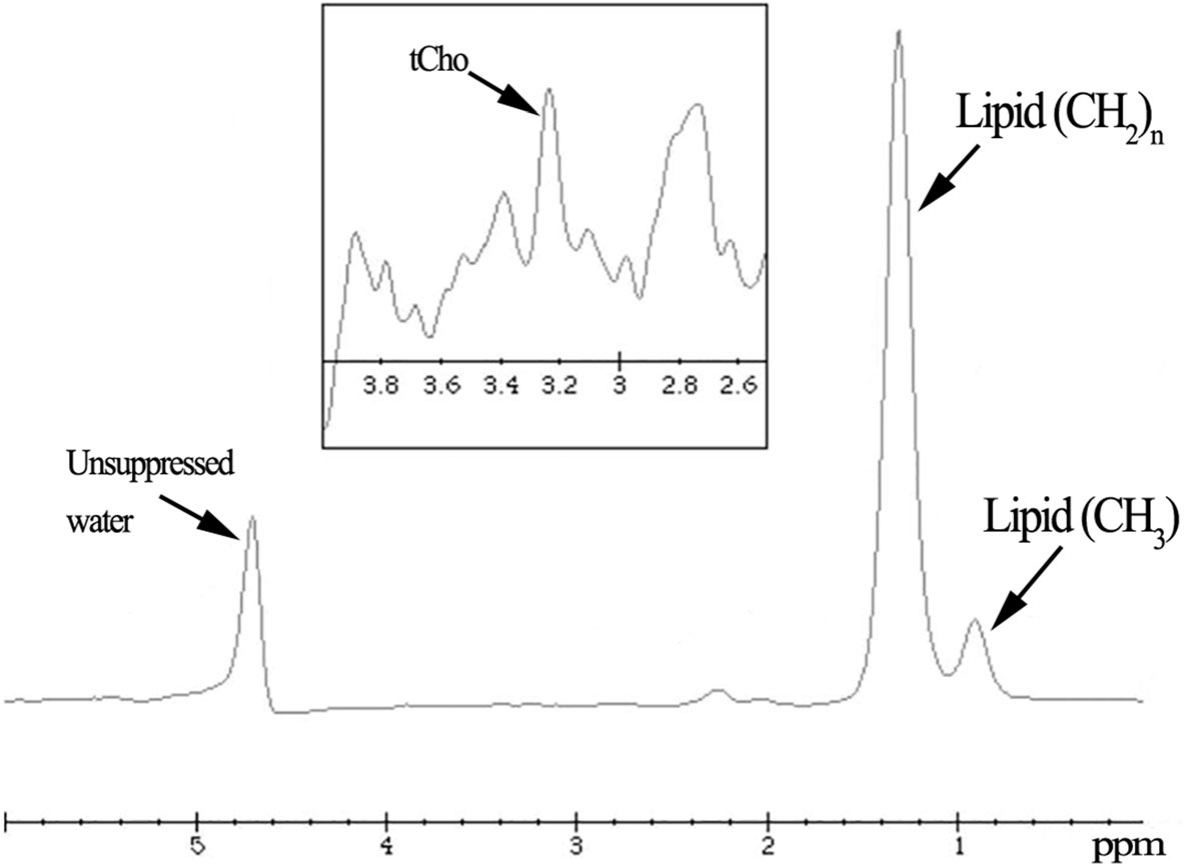
^1^H-MRS of the breast gland in a healthy fertile 26-year-old woman using oral contraceptives. A clear peak of choline-containing compounds (tCho) is visible at about 3.2 ppm, giving an absolute concentration of 0.1 mM Three main resonances are visible at about 0.9 ppm and 1.2 ppm due to lipids as well as at about 4.7 ppm due to unsuppressed water

**Fig. 3:**
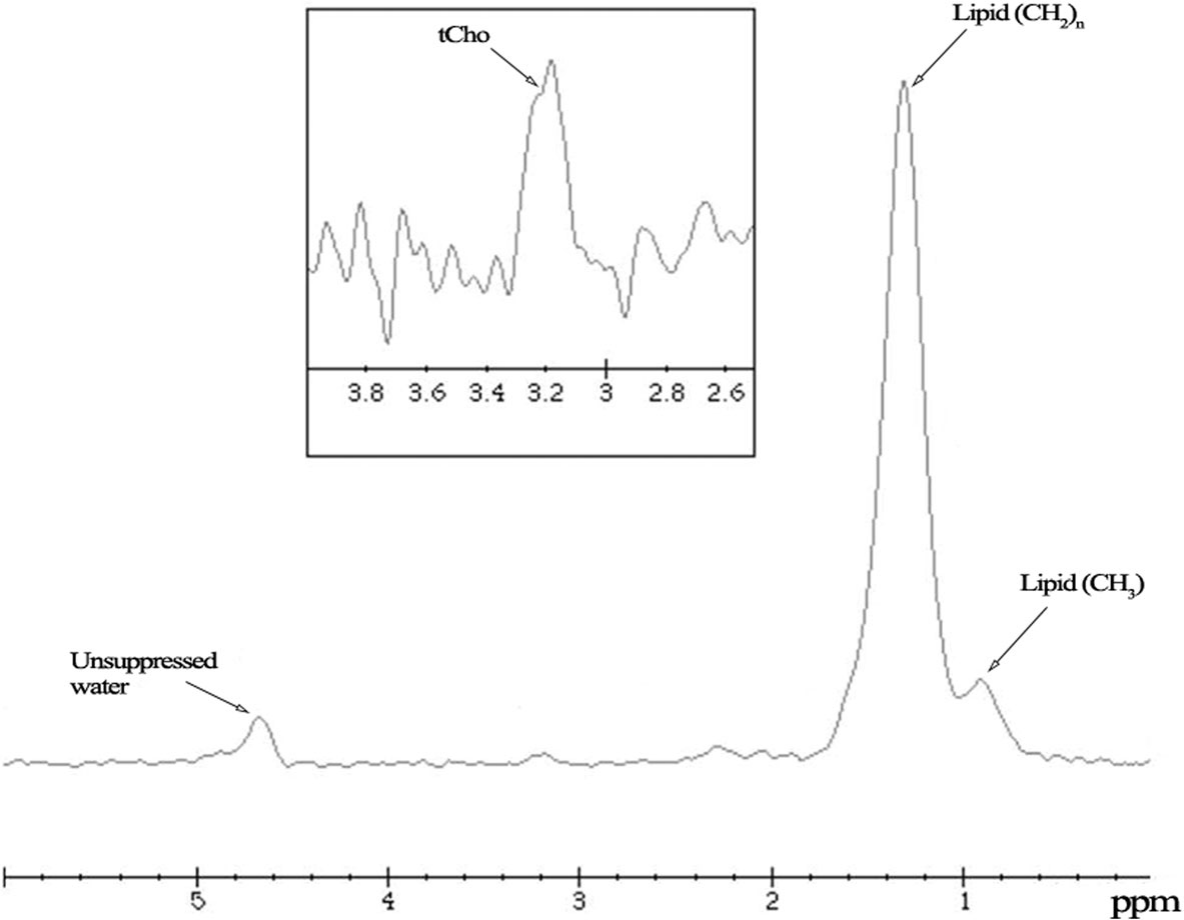
^1^H-MRS of the breast gland in a healthy fertile 24-year-old woman not using oral contraceptives. A clear peak of choline-containing compounds (tCho) is visible at about 3.2 ppm, giving an absolute concentration of 0.6 mM. Three main resonances are visible at about 0.9 ppm and 1.2 ppm due to lipids as well as at about 4.7 ppm due to unsuppressed water

None of the examinations were interrupted and all acquired spectra were of good quality. An example of ^1^H-MRS obtained in a woman from the OC group is shown in Fig. 2: a clear tCho peak was visible at about 3.2 ppm, for an absolute [tCho] of 0.1 mM. Another example of a woman in the nOC group is shown in Fig. 3, for an absolute [tCho] of 0.6 mM. This sample shows that tCho can be detected and quantified in normal breast tissue at 3 T. The spectrum obtained in the phantom led to *k*_*sys*_ of 3.4 × 10^-6^ mmol/au.

In the nOC group, [tCho] did not change significantly during the menstrual cycle. The median [tCho] in the nOC group was 0.8 (0.3–2.4) mM on day 7, 0.9 (0.4–1.2) mM on day 14, and 0.4 (0.2–0.8) mM on day 21, the differences being not statistically significant (*p* = 0.963). Per comparison, the [tCho] in the OC group (measured once) was 0.7 (0.2–1.7) mM. The difference between the [tCho] measured in the two groups was not statistically significant for all days (*p* ≥ 0.411).

These data and distributions of hormones are shown in Table 1. All hormones except prolactin showed variations during the menstrual cycle (*p* ≤ 0.024).

**Table 1:** Distributions of total choline-containing compounds (tCho), estradiol, progesterone, follicle stimulating hormone (FSH), luteinising hormone (LH), and prolactin in the group of women not using oral contraceptive (nOC) and in the group of women using oral contraceptive (OC)

	OC group	nOC group			
		Day 7	Day 14	Day 21	*p* value*
tCho (mM)	0.7 (0.2–1.7)	0.8 (0.3–2.4)	0.9 (0.4–1.2)	0.4 (0.2–0.8)	0.963
Estradiol (pg/mL)	30 (26–42)	74 (55–112)	136 (91–188)	152 (129–196)	0.024
Progesterone (ng/mL)	0.8 (0.5–1.0)	0.5 (0.4–0.8)	1.1 (0.7–2.1)	10.3 (1.6–16.3)	< 0.001
LH (mIU/mL)	0.8 (0.3–1.7)	2.2 (2.1–3.1)	4.7 (3.3–10.0)	1.9 (0.8–5.1)	0.006
FSH (mIU/mL)	2.4 (0.9–4.2)	5.7 (4.9–8.1)	7.5 (5.5–11.0)	3.6 (2.3–5.2)	0.002
Prolactin (ng/mL)	16.7 (14.1–22.3)	14.8 (14.0–17.5)	16.5 (13.2–20.0)	14.8 (12.6–18.1)	0.541

Data are medians and interquartile intervals. In the nOC group, three measurements were made on days 7, 14, and 21 of the menstrual cycle

*This comparison was made among the three distributions measured in the nOC group as a variation during the menstrual cycle

### Correlation analysis

In the OC group, [tCho] showed a borderline positive correlation only with estradiol (*r* = 0.458, *p* = 0.056); no correlation was found with other hormones (*p* ≥ 0.128). In the nOC group, [tCho] negatively correlated with prolactin (*r* = -0.587, *p* = 0.006) but only on day 7, while a positive correlation was also found with estradiol only on day 14 (*r* = 0.679, *p* = 0.001).

## Discussion

Most of the previous studies using 1.5-T magnets reported no detectable tCho levels in normal breast gland, except in the case of breast feeding or benign lesions [6, 23, 24]. Higher magnetic field strengths increase the signal-to-noise ratio and spectral resolution of ^1^H-MRS, thus allowing for a more accurate and more sensitive metabolite quantification as well as use of smaller acquisition voxels [25]. Indeed, using a 3-T magnet we were able to detect and quantify tCho in normal breast gland of young volunteers.

The most important result of this study is the demonstration of a nearly constant absolute [tCho] during the menstrual cycle in women not using OC. This concentration ranged from 0.4 to 0.9 mM without significant temporal variations. During the menstrual cycle, breast gland undergoes cyclic changes culminating in apoptosis. Mitotic activity is higher during the luteal phase because of a synergic role of both estradiol and progesterone with increases in apoptotic levels on day 28, when hormonal levels decrease. Vogel et al. [16] identified five specific phases on the basis of morphologic changes in the mammary stromal and epithelial components: proliferative (days 3–7), follicular phase of differentiation (days 8–14), luteal phase of differentiation (days 15–20), secretory (days 21–27), and menstrual (days 28–2). According to these findings and considering that tCho is a marker of metabolic turnover of phospholipids in cell membranes, we might expect different [tCho] levels between the first and the second half of the menstrual cycle. However, we did not observe any significant difference in [tCho] during the menstrual cycle, although we acknowledge that a higher statistical power, meaning a larger sample, could perhaps demonstrate some variation.

The median [tCho] measured in nOC women (0.4–0.9 mM) substantially overlaps with that obtained by Bolan [22] using internal referencing on a 4-T magnet, who reported a value of 0.66 ± 0.06 mM (mean ± standard deviation) in five healthy volunteers. The data reported by Bolan [22] fall in the middle of our interval together with a small standard deviation, but we should consider that Bolan’s study did not consider the menstrual phase. Thus, it is possible that they did not observe the full spectrum of possible tCho concentrations. Anyway, the close similarity of our and Bolan’s results is an indirect confirmation of the validity of the quantification method that we used.

Another result of this study is the demonstration of a no or negligible impact of OC on [tCho]. In fact, the median [tCho] observed in the OC group falls within the range of values obtained for the nOC group. This finding is in line with another result of this study, that is, the lack of a clear correlation between [tCho] and hormonal levels, apart from some sporadic correlations. In practice, OC impacts on the hormonal status but changes in hormonal levels do not produce significant variations in [tCho].

This pilot study has some limitations. First, ^1^H-MRS was performed only once in the OC group, under the assumption that [tCho] was constant over the menstrual cycle due to the OC mechanism of ovulation inhibition, which is responsible for stable hormonal levels. Second, even for volunteers who had a regular menstrual cycle, we could not plan MRS examinations on day 1 of the cycle. Thus, these data were missing, preventing us from deeper investigation. Third, as already mentioned, the sample size was small, likely preventing us from observing more subtle variations in [tCho] over the menstrual cycle. A larger study might give deeper insights into this issue. Fourth, we did not correct tCho peak for the coil load and the spatial variation of the coil sensitivity, but we assume that this does not represent a bias.

In conclusion, a tCho peak can be well detected by 3-T ^1^H MRS in the normal mammary gland. The [tCho] in healthy volunteers is of the order of 0.4–0.9 mM, without significant dependence on the phase of the menstrual cycle and, importantly, on the use of OC.

## Abbreviations

^1^H-MRS: Proton magnetic resonance spectroscopy
MRI: Magnetic resonance imaging
nOC: No OC group
OC: Oral contraceptives
tCho: Total choline
